# Lessons in Pysical Diagnosis

**Published:** 1868-09

**Authors:** 


### BIBLIOGRAPHICAL NOTICE.

*Lessons in Pysical Diagnosis*—By Alfred L. Loomis, M. D., Professor of the Institutes and Practice of Medicine in the University of New York; Physician to Bellevue and Charity Hospital etc. Published by Robert M. De Witt, New York.

Prof. Loomis has in this compend supplied a want long felt by the medical practitioner, and more especially by the medical student. There is no part of the practice of medicine more important, and at the same time more difficult of comprehension, than that relating to physical diagnosis; and we may safely assert that there are few graduates who at the time they receive their degree, understand perfectly the methods to be employed for detecting disease during life by the anatomical changes which it has produced.

The work consists of fifteen "Lessons" relating to the Lungs, Heart and Thoracic Aorta and Abdomen, in which the following subjects are treated in a plain and comprehensive manner: Topography of the Walls of the Chest, Contents of the various Regions, Inspection, Palpation, Mensuration and Succussion, Percussion, Auscultation, Abnormal Sounds, Auscultation of the Voice, Physical signs in the Diagnosis of Pulmonary Diseases, Topography of the Heart and Aorta, Physiological Action of the Heart, Methods of Cardiac Physical Examination, Abnormal Sounds of the Heart. Physical Signs of Pericarditis, Hypertrophy, Dilatation and Fatty Degeneration of Heart, and Aneurism of Thoracic Aorta, Topography of the Abdomen, Percussion, Palpation and Auscultation, Diseased conditions of the Peritoneum, Physical Signs of the Abnormal changes in the Different Abdominal Organs.

